# The Epicardium and the Development of the Atrioventricular Junction in the Murine Heart

**DOI:** 10.3390/jdb2010001

**Published:** 2014-03-04

**Authors:** Marie M. Lockhart, Aimee L. Phelps, Maurice J. B. van den Hoff, Andy Wessels

**Affiliations:** 1 Department of Regenerative Medicine and Cell Biology, Medical University of South Carolina, 173 Ashley Avenue, Charleston, SC 29425, USA; lockharm@musc.edu (M.M.L.); phelpsal@musc.edu (A.L.P.); 2 Academic Medical Center, Heart Failure Research Center, Department of Anatomy, Embryology and Physiology, Meibergdreef 15, 1105AZ, Amsterdam, The Netherlands; m.j.vandenhoff@amc.uva.nl

**Keywords:** heart, cardiovascular, epicardium, atrioventricular, annulus fibrosus, sulcus, valves, leaflets

## Abstract

Insight into the role of the epicardium in cardiac development and regeneration has significantly improved over the past ten years. This is mainly due to the increasing availability of new mouse models for the study of the epicardial lineage. Here we focus on the growing understanding of the significance of the epicardium and epicardially-derived cells in the formation of the atrioventricular (AV) junction. First, through the process of epicardial epithelial-to-mesenchymal transformation (epiEMT), the subepicardial AV mesenchyme is formed. Subsequently, the AV-epicardium and epicardially-derived cells (EPDCs) form the annulus fibrosus, a structure important for the electrical separation of atrial and ventricular myocardium. Finally, the AV-EPDCs preferentially migrate into the parietal AV valve leaflets, largely replacing the endocardially-derived cell population. In this review, we provide an overview of what is currently known about the regulation of the events involved in this process.

## 1. Development of the Atrioventricular Junction; a Short Introduction

### 1.1. Remodeling of the Myocardial AV Junction and Formation of the Annulus Fibrosus

The heart develops from two populations of cardiogenic anterior lateral plate mesodermal cells, known as the First Heart Fields (FHFs), which join at the ventral embryonic midline to form the primary heart tube (reviewed in [1]). This tube is comprised of three layers; an outer layer of primary myocardium, a middle layer of extracellular matrix-rich cardiac jelly, and an inner layer of endocardial cells which lines the lumen of the heart [2]. At the tubular stage, blood and solutes are propelled throughout the embryo as a result of peristaltic contraction of the primary myocardium. This primary myocardium is characterized by a low rate of proliferation and slow conduction of the cardiac impulse [3]. As development progresses the heart tube increases in length through the addition of cells from the Second Heart Field (SHF) to its arterial and venous poles. At the arterial pole this results in the formation of the outflow tract (OFT) and right ventricle [4–6], while at the venous pole the SHF contributes to atrial structures and the formation of the dorsal mesenchymal protrusion (DMP) [7–9]. As the heart is undergoing looping, expansion (or “ballooning”) of the highly proliferative atrial and ventricular myocardium results in the formation of the atria and ventricular chambers [10]. This chamber myocardium is characterized by the expression of “chamber specific” genes and typically by fast conduction of the cardiac impulse [10,11]. The myocardium of the OFT and AV canal remains, however, primary in nature. The unique characteristic of the primary myocardium of the AV canal is critically important in the early stages of heart development; the slow-conducting properties of the AV junctional myocardium delay the propagation of the cardiac impulse, generated in the atrium, allowing for the sequential contraction of atria and ventricles [12]. Later in development, this function is taken over by the annulus fibrosus, a fibrous sheath of tissue forming a physical barrier between atrial and ventricular working myocardium, thereby preventing ventricular pre-excitation and cardiac arrhythmias [13,14]. In the developed 4-chambered heart, the only place where atrial and ventricular myocardium remain in continuity, and where the cardiac impulse is propagated from the atria to the ventricles, is the central AV conduction axis consisting of the AV node and AV (or His) bundle. If the separation of atrial and ventricular working myocardium is incomplete, and accessory myocardial pathways within the annulus fibrosus allow electrical connectivity between the atrial and ventricular tissues, severe and life-threatening arrhythmias can develop, such as for instance seen in patients with Wolff-Parkinson-White (WPW) syndrome [15]. Thus, the formation of the annulus fibrosus is an important and critical event in the development of the AV junction [13,14].

### 1.2. Formation of the AV Valves

The AV valves are crucial structures in the developed 4-chambered heart. When properly formed, they prevent retrograde flow of blood from ventricles to atria during ventricular systole. The first step in AV valve development is the formation of the AV cushions. The AV cushions initially form as a result of an accumulation of cardiac jelly in the AV canal and subsequently become populated by endocardially-derived mesenchymal cells as the result of endocardial epithelial-to-mesenchymal transformation (or endoEMT) [2]. The two “major AV cushions” (the inferior and superior cushion) are the first to form [14,16]. They will eventually fuse with the mesenchymal cap of the primary atrial septum and the dorsal mesenchymal protrusion (DMP) to form the AV mesenchymal complex [17]. This complex contributes to the partition of the atrial and ventricular cavities and plays a role in the formation of the AV valve leaflets that are associated with the AV septal structures, *i.e.*, the septal leaflet of the tricuspid valve and the aortic (or anterior) leaflet of the mitral valve [17,18]. In addition to the major cushions, two smaller AV cushions form at the lateral AV myocardial junctions. These “lateral AV cushions” (right and left) develop around the time the superior and inferior cushions are fusing and, like the major cushions, also become populated with mesenchymal cells as a result of endoEMT. The right lateral cushion will eventually give rise to the parietal leaflet of the tricuspid valve, while the left lateral cushion contributes to the parietal (or mural/posterior) leaflet of the mitral valve. For consistency, throughout this review, the AV valve leaflets that derive from the right and left lateral cushions will be referred to as the parietal leaflets.

## 2. The Role of the Epicardium in AV Junction Development

### 2.1. The Epicardium in the Mouse

While there are many aspects to the development of the AV junction, in this contribution we specifically focus on the role of the epicardium and epicardially-derived cells (EPDCs) in this process. We will refer to the EPDCs at the AV junction as AV-EPDCs. The AV-EPDCs derive from the AV-epicardium, which, just like the rest of the epicardium, with the exception of the epicardium found on the outflow tract [19,20], originates from the proepicardium [21–23]. Until a few years ago, our knowledge of the importance of the epicardium in relation to the formation of the coronary vasculature, cardiac fibroblasts, and AV junction was largely based on experimental studies using avian model systems [24–27]. More recently, however, our insight into the role of the epicardium in heart development has been significantly advanced by studies in the murine heart. In the mouse, the development of the epicardium starts around embryonic day 9.5 (ED9.5) when the pro-epicardium can be identified as a villous, or “cauliflower-like”, mesothelial structure located at the venous pole of the heart [23,28]. Around ED10, cells from the proepicardium attach to the myocardial surface and begin to migrate onto the surface of the developing heart tube at the dorsal side from where the epicardium spreads over the rest of the heart, ultimately covering its entire surface [29–32].

### 2.2. “Epicardial-Cre” Mouse Models

**Figure 1:**
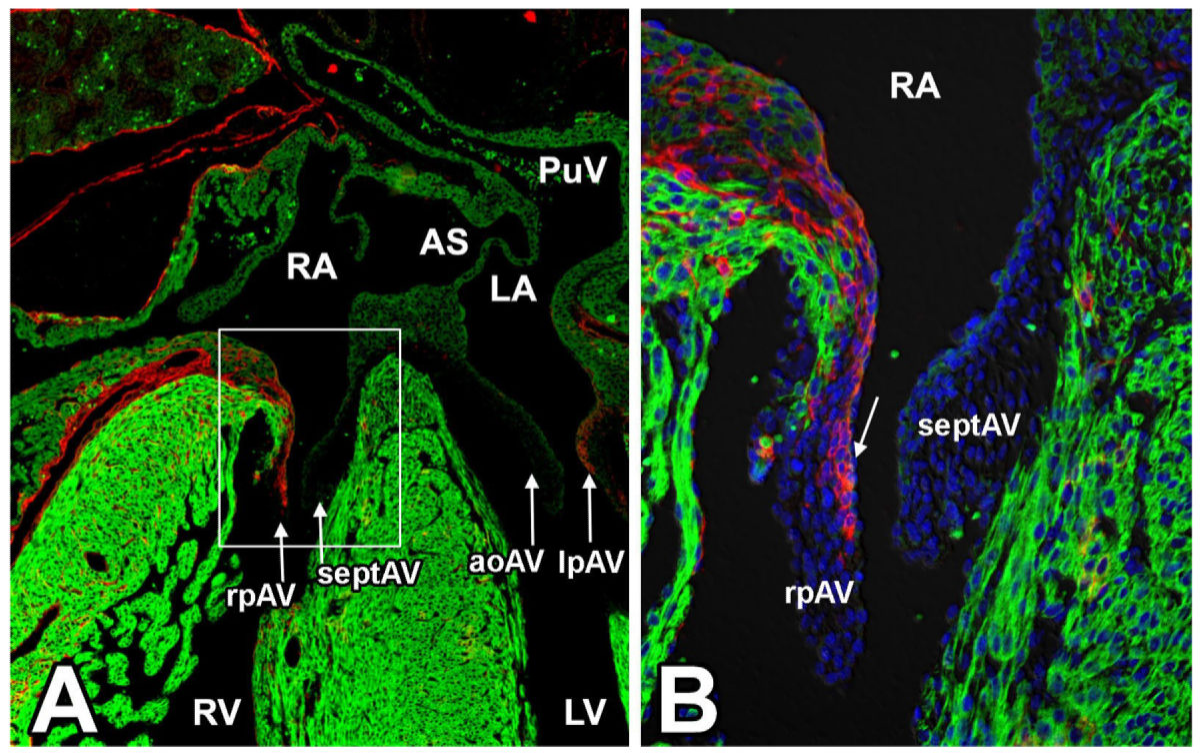
Epicardially-derived cells contribute to the parietal leaflets of the atrioventricular (AV) valves. This figure shows a transverse section of a 17ED mouse heart from a cross between a male Wt1^Cre^ mouse and a female R26^mT/mG^ reporter mouse. This cell-lineage tracing model shows that the epicardially-derived cells (in red) at the AV junction (AV-EPDCs) preferentially contribute to the right parietal (rpAV) and left parietal (lpAV) leaflets of the AV valves in a mouse heart at 17 embryonic days of development. The myocardial structures are stained for the myosin heavy chain with MF20 (green), the section was counterstained with DAPI (blue in B) to stain all nuclei. AoAV = aortic leaflet of left AV valve; AS = atrial septum; LA = left atrium; LV = left ventricle; lpAV = left parietal AV valve leaflet; PuV = pulmonary vein; RA = right atrium; RV = right ventricle; rpAV = right parietal AV valve leaflet; septAV = septal leaflet of right AV valve.

Our understanding of the role of the epicardium in heart development has been greatly advanced by the development and use of mouse models in which cre-recombinase is expressed in epicardial and/or epicardially-derived cells. These “epicardial-cre” mice include the cGATA5^Cre^ [33], the Tbx18^Cre^ [34], the inducible Wt1^CreERT2/+^ [32], the inducible Tcf21^Cre^ mouse [35], the ScxGFPCreBAC mouse [36], and the Sema3D^eGFPcre^ mouse [36]. Studies with these mouse models have primarily focused on the possible contribution of EPDCs to the coronary smooth muscle, the coronary endothelium, the interstitial fibroblast population, and to the myocardium in the developing mouse heart [32,33,35,37–39]. Recently, we reported the use of a mWt1/IRES/GFP-Cre (or Wt1^Cre^ in this paper) mouse which, in combination with the R26^mT/mG^ reporter mouse, we used to conduct epicardial-lineage tracing experiments, specifically focusing on the spatiotemporal contribution of the epicardium to the development of the tissues at the AV junction [29]. We determined that, after the formation of the AV sulcus and the annulus fibrosus, a subset of AV-EPDCs migrate into the derivatives of the AV cushions. Specifically, the EPDCs populate the derivatives of the lateral AV cushions, but not the derivatives of the major AV cushions (Figure 1). A more detailed description of this process is provided below.

### 2.3. Cascade of Events Involved in Epicardial Contribution to AV Development

**Figure 2:**
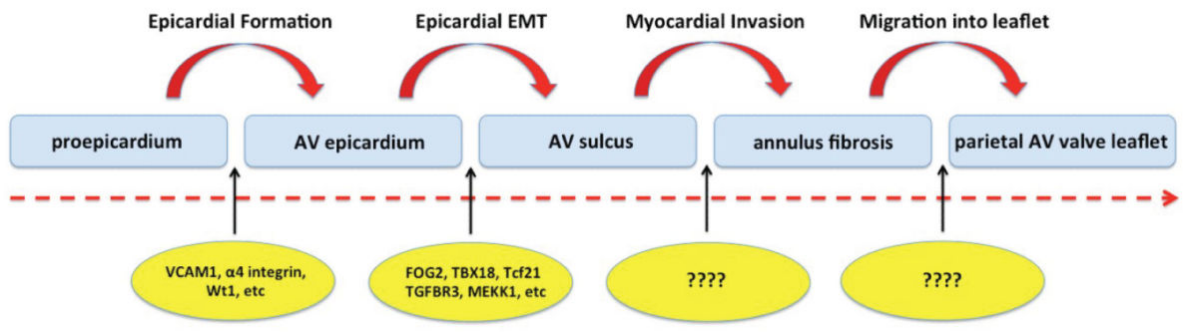
Cascade of events involving the epicardial and epicardially-derived cells in the development of the AV junction. The epicardium plays a crucial role in virtually all aspects of the development of the structures at the AV junction. While insight into the molecular mechanisms that control the formation of the epicardium proper as well as epicardial EMT is steadily growing, virtually nothing is known about the molecular mechanisms that govern the formation of the annulus fibrosus and the ones that are involved in the migration of AV-EPDCs into the parietal AV valve leaflets.

As the epicardium proper is forming, the tissues at the AV junction are undergoing significant remodeling. Whereas at the interior side of the AV junction, endoEMT is responsible for the generation of the endocardially-derived mesenchyme of the AV cushions, the epicardial EMT (epiEMT) taking place at the exterior AV junction (or AV groove) is responsible for the accumulation of epicardially-derived mesenchyme in the AV sulcus. Subsequently, a subpopulation of these AV-EPDCs starts to penetrate the lower boundary of the AV canal myocardium, resulting in the formation of the annulus fibrosus and in the incorporation of the primary AV canal myocardium into the atrial chambers [14]. Eventually, the AV-EPDCs reach and populate the parietal leaflets of the AV valves [14,29,37]. The migration of AV-EPDCs into the AV junction, the formation of the annulus fibrosus, and the subsequent migration of the AV-EPDCs into the parietal AV valve leaflets also define the hinge point for these AV valve leaflets. In the following sections we will briefly discuss aspects of what is currently known about the involvement of the epicardium in this series of events. Based on the studies and observations described in this contribution, we have put together a simple model reflecting the cascade of epicardial-associated events involved in the remodeling of the embryonic AV junction (Figure 2). In this model we identify essentially four critical steps in the process. How these steps are regulated and controlled is the focus of ongoing studies.

## 3. Contribution of EPDCs to the AV Junctional Tissues

### 3.1. Formation of the AV Epicardium

The first step in the series of epicardial-related events that regulate the remodeling of the AV junction is the formation of the AV epicardial epithelium (or AV epicardium) itself. This process is initiated when cells from the proepicardium reach the myocardial surface of the heart around ED9.5. For many years it was believed that the mechanism through which this is achieved is fundamentally different between the mammalian and the avian heart. Specifically, it was thought that while in the avian heart the proepicardium is making contact with the heart proper via protruding proepicardial villi, the primary mechanism of how the proepicardial cells reach the heart in the mammalian heart is by the release of proepicardial vesicles into the pericardial cavity that subsequently “land” on the myocardial surface to form the epicardium [40,41]. More recently, however, data have been presented that strongly suggest that the translocation of proepicardial cells to the myocardial surface of the mammalian heart may actually be very similar to that of the avian heart and in fact involves the expansion of multicellular villi from the proepicardium toward the atria and ventricles of the beating heart [42]. Interestingly, the paper by Rogers and colleagues reports that these proepicardial projections connections are not seen between the proepicardium and the AV junction but that, instead, proepicardial vesicles (or cysts) are found in the AV groove. The authors propose that the vesicles observed in the AV groove possibly are segments of proepicardial villi that detach from these projections as a result of dynamic fluid force. It can, however, not be excluded that the proepicardial vesicles that specifically attach to AV structures might actually represents an alternative AV-specific mechanism for epicardium formation. Irrespective of the mechanism that is responsible for the initial attachment of epicardial cells to the myocardial surface of the early embryonic heart, the epicardium eventually spreads as an epithelial sheet over the rest of the heart. Over the years, a series of molecular mechanisms have been identified that play a role in the establishment of the epicardium proper. Genes involved in these mechanisms include alpha4 integrin [43], VCAM1 [44], and Wt1 [31].

### 3.2. Epicardial EMT at the AV Junction and Formation of the AV Sulcus

The second step in the cascade of epicardial-related events at the AV junction is the formation of epicardially-derived cells forming the mesenchyme of the AV sulcus (or AV-EPDCs). This process is initiated by epicardial epiEMT and is followed by the expansion of the AV-EPDC population by proliferation. The AV-EPDCs in the AV sulcus form an important pool of precursor cells for the development of a number of structures at the AV junction. Not only do the AV-EPDCs play a critical role in the formation of the annulus fibrosus and contribute to the development of the parietal AV valve leaflets [29], the AV-EPDCs also are involved in the formation of the coronary vasculature that develops within the AV sulcus tissue. Although we know relatively little about epiEMT, certainly compared to what is currently understood about the molecular mechanisms that control endocardial endoEMT, insight into how epiEMT is regulated is slowly emerging [45–48]. In the following sections we will specifically focus on epiEMT at the AV junction.

**Figure 3:**
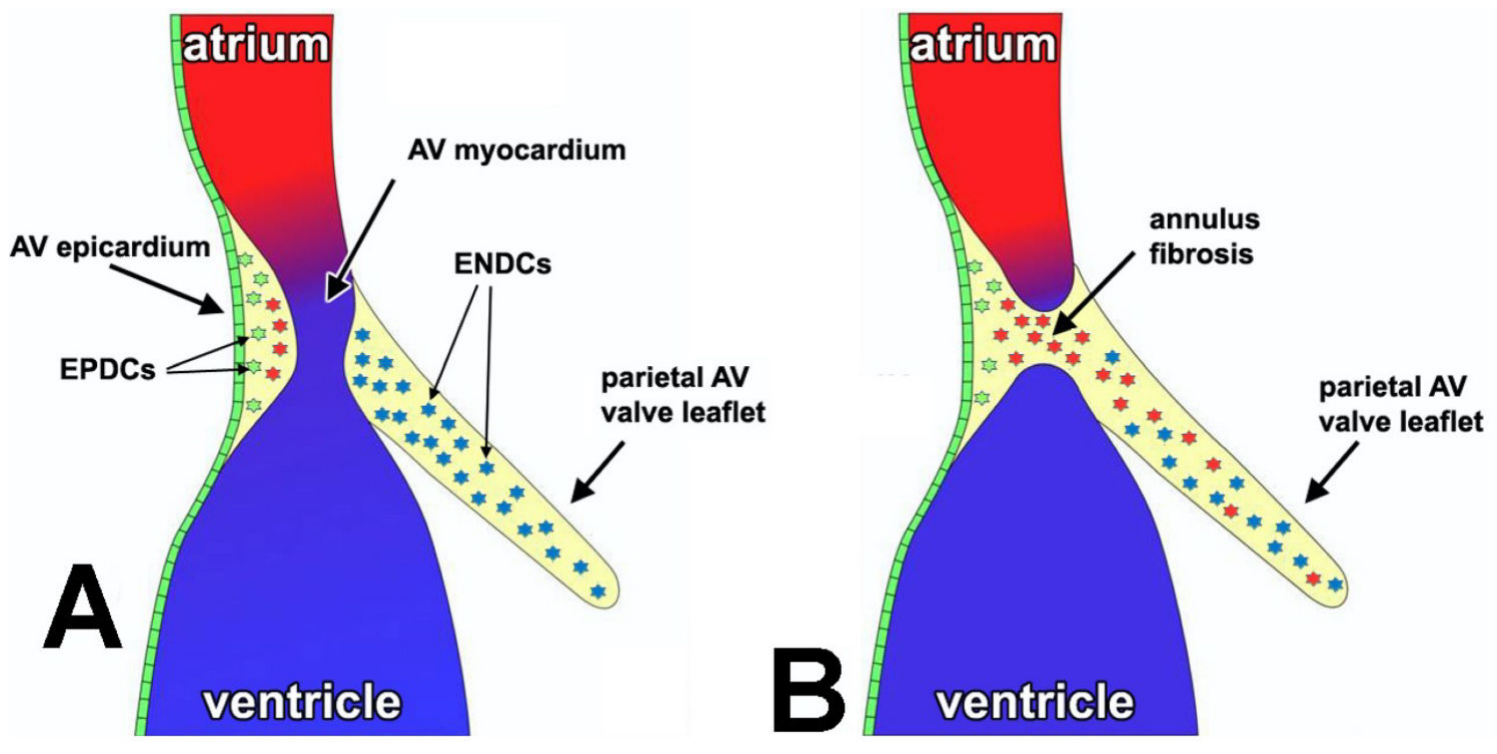
Schematic representation of the contribution of the AV epicardium and the epicardially-derived cells to the development of the AV junction. After formation of the epicardial epithelium (green), epiEMT generates a population of AV-EPDCs (green cells in panel A) that, as far as their gene expression profile is concerned, are still very similar to the epicardium itself. However, when the AV-EPDCs migrate further into the AV sulcus and approach the AV myocardium (red cells), the molecular profile of the AV-EPDCs changes drastically as the expression of genes characteristically found in the mesenchyme of the annulus fibrosus (e.g., MMP2) and the AV cushions (e.g., Sox9) is upregulated. These “differentiated” AV-EPDCs (red cells in panel A) then penetrate the AV myocardium to form the annulus fibrosus (panel B) and migrate into the parietal AV valve leaflets where they intermingle with the endocardially-derived mesenchymal cells (ENDCs; blue cells in panels A and B).

It is well recognized that a number of genes expressed at relatively high levels in the AV junctional myocardium are important in the regulation of endoEMT in the AV cushions. In particular the roles of Tgfβ2 and Bmp2 are well recognized in this process [49,50]. Given the fact that the AV sulcus is situated at the opposite side of the AV junctional myocardium, it is therefore not surprising that these growth factors are also implicated in the regulation of epiEMT. Thus, it has been demonstrated that Tgfβ2 can induce epiEMT in epicardial explants from chick embryos through activin receptor-like kinase 5 (ALK5) [51], while experiments with immortalized epicardial cells suggest that BMP2 can stimulate epiEMT. AV epicardial cells express, just like epicardial cells that cover other parts of the heart, markers such at Tbx18 and Tcf21. They also express the transcription factor Wt1 that has been associated with the direct transcriptional regulation of epiEMT through multiple downstream targets. Wt1 expression in cultured epicardial cells promotes migration by activating transcription of the cell motility-associated gene Coronin 1b and the EMT-inducing transcription factor Snail/Snai1, as well as by repressing transcription of the cell-adhesion associated gene E-cadherin [52,53]. Wt1 also regulates epiEMT through activation of Raldh2, an enzyme which catalyzes the dehydrogenation of retinaldehyde to retinoic acid and which is an important morphogen in the developing heart [54–56]. As mentioned, Tgfβ2, expressed at high levels in the AV junctional myocardium, is an established activator of endoEMT. Tgfβ2 can also stimulate hyaluronan synthesis in epicardial cell culture [57]. Hyaluronan is a component of the extracellular matrix (ECM), synthesized by Hyaluronan Synthase 2 (Has2) and found to be essential for endoEMT. Because mice that are unable to synthesize hyaluronan (Has2 null) die before the establishment of the epicardium and the formation of the AV sulcus, this particular mouse model did not provide much insight into the specific importance of HA in the regulation of epicardial cell behavior at the AV junction [58]. However, *in vitro* studies with epicardial cells have demonstrated that the high molecular weight form of HA (HMW-HA) is able to initiate epiEMT by binding to the HA receptor Cd44, present on epicardial cells, and activating Mekk1 binding to CD44 [59]. Mekk1 signals through Erk and NFκB, promotes epiEMT, and the expression of vimentin [60], an intermediate filament gene upregulated as the AV-EPDCs migrate away from the epicardium toward the AV junctional myocardium (Figure 3).

Previously, it has been described that when ventricular EPDCs migrate into the ventricular wall they gradually lose expression of Wt1 and Raldh2 and that they gain expression of mesenchymal cell markers such as Sox9 [61], Snail/Snai1 [62], and Slug/Snai2 [47,63]. This same change in gene expression is also observed at the AV junction where Wt1 and Raldh2 are expressed in the AV epicardial cells and in the subepicardial AV sulcus mesenchyme [27]. In our studies, we observe a shift in the molecular profile of AV-EPDCS as they are migrating away from the epicardium and approaching the AV myocardium. While they are gradually losing their Wt1 expression, immunohistochemical staining for the transcription factor Sox9, a gene characteristically expressed at high levels in mesenchymal cells and an important transcriptional regulator of extracellular matrix (ECM) in developing cardiac valves [29,61,64,65] becomes more intense. Another gene found to be upregulated as the AV-EPDCs approach the AV myocardium is matrix metalloproteinase Mmp2. Taken together, the changes in gene expression that are observed in the AV-EPDCs reflect their ongoing differentiation into a more fibroblast-like phenotype as they move deeper into the AV sulcus and approach the AV myocardium. As the AV-EPDCs start to invade the AV junctional myocardium they are, based on our current insights, phenotypically similar to the AV-EPDCs that subsequently will form the annulus fibrosus and migrate into the parietal leaflets.

### 3.3. The Annulus Fibrosus

In studies using the Wt1^CreERT2/+^ mouse in conjunction with the Rosa26^mT/mG^ reporter mouse, the Pu lab was the first to unambiguously show that EPDCs contribute to the formation of the annulus fibrosus in the mammalian heart [37]. Using immunohistochemical analysis, they demonstrated that EPDCs at the AV junction express two important ECM components of the annulus fibrosus, namely collagen I and periostin [37]. In addition, qRT-PCR analysis of GFP^+^ cell-sorted AV-EPDCs from the AV junction showed that EMT-associated genes, fibroblast markers, and the matrix metalloproteinase Mmp2 are enriched in these cells [37]. Further comparisons between EPDCs of the AV junctional and EPDCs from the apical ventricular region of the embryonic heart showed that AV junctional EPDCs are enriched for EMT markers such as Snail, Slug, and Twist1 as well as for fibroblast markers such as periostin, tenascin C, and discoidin domain receptor 2 (Ddr2), indicating that EPDCs that contribute to the AV junction (AV-EPDCs) have different characteristics than EPDCs that invade the myocardial wall [37]. Thus, this study provided novel insights into the role of the epicardium in the formation of the annulus fibrosus. In earlier studies, *i.e*., well before we had developed an understanding of the role of the epicardium in AV development, we concluded that, based on immunohistochemical and morphological observations in the developing human heart, the annulus fibrosus typically forms at the lower boundary of the AV junctional myocardium and that, as a result, the original AV junctional myocardium eventually becomes incorporated in the lower rim of the atrial chambers [14,66]. The (molecular) mechanism that determines the “point of entry” for the invading AV-EPDCs has not been determined yet. It is important to note that separation of atrial and ventricular myocardium is not taking place in the posterior wall of the heart. Although a well-defined AV sulcus does develop here, and AV-EPDCs do migrate into the myocardium, the atrial and ventricular myocardium does not get separated. This is very important, as the resulting “persisting” myocardial AV connection form the proximal part of the AV conduction system (*i.e*., the AV node and AV bundle) [66].

### 3.4. EPDCs in the Leaflets of the AV Valves

The role of the epicardium in the development of the AV valve leaflets has been an area of interest for years. In a 1986 paper on the formation of the mitral valve [67], it was suggested that ingrowth of the tissues of the AV groove (which we now know consist of AV-EPDCs) was not only responsible for the formation of the annulus fibrosus, but that the material of the AV groove also formed the AV leaflets. The authors proposed a mechanism in which the tissue of the AV groove pushed through the AV junctional region thereby displacing the endocardially-derived mesenchyme toward the tip of the AV valve leaflet. These remnants, according to the authors, could be recognized in the developed heart as the “noduli albini” [67]. In later studies on the developing human heart, we revisited this controversial topic using an immunohistochemical approach and did not find any evidence in support of this model [14]. Instead, based on our analysis, we determined that the tissues of the AV groove and AV cushions fuse at the luminal side of the AV junction and therefore concluded that “the tissues of the atrioventricular groove do not contribute to the development of these (AV) leaflets” [14]. Based on more advanced cell fate studies performed in recent years we now understand that the truth lies somewhere in the middle. Below we will review what is currently known about the contribution of the epicardium to the developing AV valve leaflets.

The initial studies on the fate of EPDCs in the developing heart using avian models strongly suggested that EPDCs were able to migrate into the AV cushions [24,25]. These studies did, however, not provide much insight into the spatiotemporal contribution of the EPDCs to the respective leaflets. In addition, the experimental approach in the quail-to-chick chimera technique, in which a proepicardial explant from a donor quail embryo is placed in the pericardial cavity of a host chicken embryo, left many questions unanswered. For instance, in a study on the lineage and morphogenic analysis of the AV valves [68] it was reported that using the quail-to-chick approach, no EPDCs could be found in the formed AV valve leaflets. This, given the reported presence of EPDCs in the developing leaflets, raised significant questions about the fate of the EPDCs at later stages of AV valve formation. Thus, we decided to revisit the role of EPDCs in AV valve formation in the mouse using molecular cell fate tracing with our Wt1^Cre^-R26^mT/mG^ mouse model system [29]. In this study we traced the fate of EPDCs throughout cardiac development focusing on their contribution to the AV valves. We found that the parietal leaflets of the AV valves, which derive from the lateral AV cushions, gradually become populated with EPDCs from ED13 onward. The AV valve leaflets that derive from the major AV cushions remain, however, largely devoid of EPDCs (see Figure 1). Based on the distribution of Wt1^Cre^-R26^mT/mG^ labeled cells, in combination with the results of endocardial-cell fate studies using the Tie2^Cre^; R26^mT/mG^ model, we concluded that in the fully developed parietal leaflets, EPDCs form the majority of the valve cells and that only a relatively small portion of mesenchymal cells in these leaflets is endocardially-derived. Importantly, the mesenchyme of the septal leaflet of the right AV valve and the aortic leaflet of the left AV valve remains mostly endocardially-derived [29,69]. The observation that the formed parietal leaflets largely consist of cells that are derived from the epicardium and that the leaflets associated with the septal structures (*i.e.*, the septal leaflet of the right AV valve and the aortic - or anterior- leaflet of the left AV valve) contain mainly cells that are derived from the endocardial lineage raises questions regarding potential differences in the functional properties of these cells.

## 4. Discussion and Future Directions

Much of what is currently known regarding the genes involved in epiEMT and EPDC contribution to the fibroblast populations of the developing heart is based on studies of the epicardium covering the ventricles [48,70]. However, it is yet to be established whether the genes that have been identified to be important in “ventricular epiEMT” are also playing an equally important role in “AV-epiEMT”. Moreover, it remains to be determined whether the EPDCs that result from these two, potentially differently regulated events, have the same characteristics. In fact, recent work suggests that AV junctional and apical/ventricular EPDCs express distinctive differentiation markers [37]. This may not be surprising as there are quite a number of growth and transcription factors (e.g., Tgfβ2, Bmp2, Tbx3) that are expressed differentially in the AV junctional myocardium and the ventricular wall [71,72]. These expression patterns are responsible for the activation and/or differential regulation of various gene programs involved in AV junction development and are likely also involved in the regulation of the formation and differentiation of epicardially-derived valve interstitial fibroblasts *versus* epicardially-derived ventricular fibroblasts. Furthermore, it is possible that intrinsic differences exist between the epicardium in the AV junction and epicardium on the ventricular wall and that this will result in different cell behavior. For example, Sox9 has been shown to be required for epicardial cell EMT and differentiation into the cardiac fibroblast lineage in response to Pdgfrα [73], however abnormalities related to the parietal leaflets in Pdgfrα knockout mice and epicardial-specific PDGFRα knockout have not been described [35,36]. Elucidating the differences between epiEMT at the AV junction and ventricular wall will be the focus of future research efforts.

The preferential contribution of EPDCs to the parietal leaflets of the AV valves presents several questions concerning the role of EPDCs in AV valve development, including: (i) what determines the migration of EPDCs into the AV valve leaflets; (ii) do the EPDCs in the parietal leaflets regulate the process that leads to the reduction of endocardially-derived cells in these leaflets; (iii) what are the similarities and differences in the molecular and functional characteristics of the epicardially- and endocardially-derived cells that reside in the AV valves; and (iv) what differences in gene expression, if any, exist between the parietal leaflets and the “septal” leaflets of the AV valves? Although we have previously established that the EPDCs in the parietal leaflets express many of the same proteins that are also expressed by the endocardially-derived valve interstitial cells in the AV leaflets (e.g., collagen I, periostin, vimentin, filamin A, and sox9 [29]), the genes investigated are only a few out of the ever-increasing number of genes identified to be important in valve development. A more comprehensive analysis, for instance following an RNAseq approach, would undoubtedly shed more insight into this question.

Finally, in addition to understanding the molecular differences in the endocardially-derived and epicardially-derived cells of the AV valves, it is important for future research to address what role the AV-EPDCs play in AV valve development and maturation. Removal of the proepicardium in avian embryos results in a spectrum of cardiac defects, including thin myocardium, abnormal outflow tract septation, failure of coronary plexus formation, failure of the AV cushions to fuse, and enlargement of the AV cushions [20,25,27]. Similarly, experiments in chicken hearts in which the epicardial cell contribution was blocked resulted in a loss of AV sulcus mesenchyme, impaired formation of the annulus fibrosus resulting in the persistence of the AV myocardial continuity and as such providing accessory pathways for the conduction of the cardiac impulse [26,74]. These results suggest that EPDCs, at least in the avian system, are required for normal AV valve development to proceed after the initial formation and growth of the cushions by endocardial EMT. Additionally, these results indicate that interactions between epicardially-derived and endocardially-derived cells occur to direct normal valve development. This mixing of cells from separate origins in the developing AV cushions is reminiscent of what happens during outflow tract development when endocardially-derived cells and neural crest derived-cells together contribute to the mesenchyme of the outflow tract ridges [75]. To fully establish the function of AV-EPDCs in the development and maturation of the leaflets of the AV valves, and to determine whether these cells have a specific role in controlling functional characteristics of the AV valves in the pre- and postnatal heart, and/or whether AV-EPDCs are involved in the pathogenesis of valve diseases, it is essential to develop (new) research strategies that allow us to address these questions.
